# Deep Sequencing for Evaluation of Genetic Stability of Influenza A/California/07/2009 (H1N1) Vaccine Viruses

**DOI:** 10.1371/journal.pone.0138650

**Published:** 2015-09-25

**Authors:** Laassri Majid, Tatiana Zagorodnyaya, Ewan P. Plant, Svetlana Petrovskaya, Bella Bidzhieva, Zhiping Ye, Vahan Simonyan, Konstantin Chumakov

**Affiliations:** Center for Biologics Evaluation and Research, US Food and Drug Administration, 10903 New Hampshire Avenue, Silver Spring, Maryland, 20993, United States of America; Icahn School of Medicine at Mount Sinai, UNITED STATES

## Abstract

Virus growth during influenza vaccine manufacture can lead to mutations that alter antigenic properties of the virus, and thus may affect protective potency of the vaccine. Different reassortants of pandemic "swine" H1N1 influenza A vaccine (121XP, X-179A and X-181) viruses as well as wild type A/California/07/2009(H1N1) and A/PR/8/34 strains were propagated in embryonated eggs and used for DNA/RNA Illumina HiSeq and MiSeq sequencing. The RNA sequences of these viruses published in NCBI were used as references for alignment of the sequencing reads generated in this study. Consensus sequences of these viruses differed from the NCBI-deposited sequences at several nucleotides. 121XP stock derived by reverse genetics was more heterogeneous than X-179A and X-181 stocks prepared by conventional reassortant technology. Passaged 121XP virus contained four non-synonymous mutations in the HA gene. One of these mutations (Lys_226_Glu) was located in the Ca antigenic site of HA (present in 18% of the population). Two non-synonymous mutations were present in HA of viruses derived from X-179A: Pro_314_Gln (18%) and Asn_146_Asp (78%). The latter mutation located in the Sa antigenic site was also detected at a low level (11%) in the wild-type A/California/07/2009(H1N1) virus, and was present as a complete substitution in X-181 viruses derived from X-179A virus. In the passaged X-181 viruses, two mutations emerged in HA: a silent mutation A_1398_G (31%) in one batch and G_756_T (Glu_252_Asp, 47%) in another batch. The latter mutation was located in the conservative region of the antigenic site Ca. The protocol for RNA sequencing was found to be robust, reproducible, and suitable for monitoring genetic consistency of influenza vaccine seed stocks.

## Introduction

The influenza A/California/07/2009 (H1N1) virus emerged in Mexico, and was identified in the United States at the beginning of 2009. It caused outbreaks of respiratory illness in Mexico and in the United States, [1] and was shown to be genetically related to swine influenza viruses [2]. Subsequently, the virus spread to several other countries in North America, and eventually the outbreak reached pandemic proportions, which was recognized by the World Health Organization (WHO) in June of 2009 [3, 4].

The quick emergence and spread of A/California/07/2009 (H1N1) virus has been of great concern globally. Therefore, the development of A /California/07/2009 vaccine was an essential component of the global preventive strategy for the pandemic preparedness and response. WHO recommended that pandemic influenza A (H1N1) vaccines must contain A/California/7/2009 (H1N1)-like virus [5].

To date, several vaccines have been developed against A/California/07/2009 (H1N1) virus. Some vaccine viruses were prepared by classical reassortment (e.g. X-179A and X-181); others by reverse genetics (e.g. 121XP). They were manufactured as seasonal influenza vaccines manufactured i.e. after virus cultivation; the allantoic fluid of the egg is harvested and is then usually clarified by centrifugation to remove cellular debris. Then whole virus subjected to purification, concentration, and inactivation steps.—See more detail at: http://www.ifpma.org/resources/influenza-vaccines/influenza-vaccines/vaccine-manufacture-virus-processing.html#sthash.5GUYGsGs.dpuf.

The A/California/07/2009 (H1N1) strain is an influenza A virus, a single-stranded RNA virus from *Orthomyxoviridae* family [6]. The family contains four other species: influenza B virus, influenza C virus, thogotovirus and isavirus. The genome of influenza A virus consists of eight negative-sense RNA segments that encode at least 10 proteins. Influenza A virus genome is highly variable due to low fidelity of RNA polymerase and reassortment between co-infecting strains [7]. New virus mutants emerge continuously, allowing viruses to evade host immunity and cause cyclical annual outbreaks, and occasionally pandemics.

Spontaneous mutations easily emerge in RNA viruses, and the consequences of their accumulation must be identified to ensure the safety and potency of vaccines. The genetic diversity within a viral population is important, as RNA viruses in particular have high mutation rates, can evolve rapidly, and may exhibit adaptation to specific hosts and/or tissues [8]. Because of the genetic and antigenic plasticity, influenza vaccines have to be reformulated frequently to include antigens of the dominant circulating strains. Most live and inactivated influenza vaccines are produced by reassortment of the current strains with high-growth reference strains, to enable high yields in eggs or cell substrates used for vaccine production [9, 10]. Adaptation to growth in different cells can lead to changes in the viral receptor-binding region, as well as in protective epitopes, because they significantly overlap each other [11, 12]. Therefore, it is important to monitor genetic changes of viruses used in vaccine manufacture to ensure that their antigenic structures remain intact.

Most mutants are present in viral populations at relatively low levels, making them difficult to detect using conventional sequencing methods. A highly sensitive mutant analysis by PCR and restriction enzyme cleavage (MAPREC) [13, 14] and quantitative allele-specific PCR (asqPCR) [15] were developed for detection of a low level of mutants in viral vaccines. However, these methods are only suitable for analysis of one mutation at a time, and cannot be used to screen for unknown mutations.

Other approaches based on analysis of electrophoretic mobility in gels [16, 17] do not always allow precise location of mutations. Matrix-assisted laser desorption/ionization-time of flight (MALDI-TOF), mass spectrometry [18], and hybridization with microarrays of short oligonucleotides [19–21] are sensitive, but are relatively laborious and may require follow-up by direct sequencing.

Recently, several versions of high-throughput sequencing technology, known also as deep or massively parallel sequencing (MPS), enabled rapid generation of large amounts of sequence information [22]. These new technologies were shown to be suitable for analysis of heterogeneities in viral populations [23]. Previously, we have demonstrated that MPS can be used to monitor genetic stability of Oral Polio Vaccines (OPV) [24], and could replace the WHO-recommended MAPREC assay for lot release of OPV. In this study, Illumina sequencing technology was used for screening mutations in viruses of A/California/07/2009 (H1N1) vaccine strains passaged in eggs. We found that this approach could be used to identify and to accurately quantify small amounts of mutant viruses present in preparations of inactivated influenza vaccines. We also found that sequence heterogeneity of seed viruses depended on the methods used for their preparation. The ability to quantify potentially undesirable mutations in vaccine batches makes this method suitable for quality control to ensure manufacture of safe and effective vaccines.

## Materials and Methods

Viruses and reference sequences

Viruses X-179A, X-181 and 121XP were passaged in eggs under similar conditions to those of the manufacturers [25, 26], and A/Puerto Rico/08/34 (H1N1) reference and A/California/07/2009 (H1N1) pandemic virus stocks (Table 1), obtained from the strain collection at the Center for Biologics Evaluation and Research, US Food and Drug Administration were grown in eggs. All these viruses (Table 1) were propagated in 10 days old embryonated hen’s eggs by inoculation of 0.2 ml of diluted virus stock containing about 10^4^ pfu at 33°C. Allantoic fluid was harvested at 72 hours post infection and clarified by spinning at 4000 rpm for 10 min at 4°C. The clarified viruses were saved at -80°C.

**Table 1:** Viruses of A/California/07/2009 vaccines, and A/PR/8/34 and wild type A/California/07/2009 viruses used for deep sequencing analysis. Note: CBER: Center for Biologics Evaluation and Research at US Food and Drug Administration.

Sample code	Reference and Derivation	Number of passages on eggs
X-181	X-181	1
X-181-M1	X-181	9
X-181-M2	X-181	9
X181-M3	X-181	13
X-181-M4	X-181	10
X-179A	X-179A	1
X-179A-M1	X-179A	10
X-179A-M2	X-179A	11
X-179A-M3	X-179A	12
X-179A-M4	X-179A	11
X-179A-M5	X-179A	13
121XP-M4	121XP	10
A/Ca/07/2009	A/California/07/2009	1
A/PR/8/34 (CBER)	A/Puerto Rico/08/34	1
A/PR/8/34-1*	A/Puerto Rico/08/34	14
A/PR/8/34-3*	A/Puerto Rico/08/34	14

*: A/PR/8/34 virus stock was passaged 13 times in eggs, and the last passaged virus was passaged one time (passage 14) separately in 2 different eggs, the 2 harvested viruses are designated as A/PR/8/34-1 and A/PR/8/34-3.

### Amplification of the entire influenza virus genome

Total RNA was isolated from 140 μl of virus suspension using QIAamp Viral RNA Mini Kit (Qiagen, Valencia, CA) according to the manufacturer’s protocol. The RNA was eluted in a final volume of 60 μl of sterile RNase-free water. cDNA was synthesized using Superscript III Reverse Transcriptase (SSIII, Invitrogen, Carlsbad, CA) with Universal_F primer: 5’ GACTAATACGACTCACTATAGGGAGCAAAAGCAGG 3’. cDNA synthesis was performed in a reaction containing 10 μl of isolated viral RNA, 1.6 μM of Universal_F primer, 600 units of SSIII, 0.5 mM dNTPs, 50 mM DTT, and first strand buffer in a total volume of 50 μl. The reaction mixture was incubated at 55°C for two hours, followed by SSIII inactivation at 70°C for 20 min.

Two different PCR protocols were used to simultaneously amplify all genome segments of influenza virus.

Phusion protocol: Four microliters of viral cDNA was used as a template for PCR amplification in a total reaction volume of 50 μl, containing 0.2 μM of each universal primers: Universal_F (see above) and Universal_R: 5’GACATTTAGGTGACACTATAGAAGTAGAAACAAGG 3’, 200 μM of dNTP, 3 mM MgCl_2_, and 1 unit of Phusion DNA polymerase (New England BioLabs, Ipswich, MA). PCR cycling conditions were as follows: an initial denaturation at 98°C for 30 sec followed by 35 cycles of 98°C for 10 sec, 55°C for 30 sec and 72°C for 5 min, and the final elongation at 72°C for 10 min.

Extensor protocol: Four microliters of viral cDNA was used as a template for PCR amplification in a total reaction volume of 50 μl containing 0.4 μM of each universal primer (Universal_F and Universal_R), 500 μM of dNTP, 5 μl of 10x Extensor Buffer 2, and 2.5 units of Extensor PCR Enzyme Mix (5U/μl) (Dharmacon, Lafayette, CO). PCR cycling conditions were as follows: an initial denaturation at 94°C for 2 min followed by 35 cycles of 94°C for 10 sec, 60°C for 30 sec and 68°C for 5 min, and the final elongation at 68°C for 10 min.

The resulting eight DNA amplicons representing all segments of viral genome were separated by electrophoresis in 1% agarose gels with ethidium bromide (Lonza, Rockland, ME) and visualized using the Kodak Gel Logic 200 Imaging System and Kodak Molecular Imaging Software (Carestream Health, Inc. Rochester, NY).

### Preparation of samples for Illumina sequencing

The PCR product was purified by QIAquick PCR Purification Kit (Qiagen, Valencia, CA) and fragmented by focused ultrasonicator (Covaris) to generate the optimal fragment sizes of 350–400 bp suitable for Illumina sequencing.

The DNA fragments were analyzed for DNA size by the 2100 Bioanalyzer system using a high-sensitivity kit (Agilent Technologies, Inc., Santa Clara, CA). Next, the fragmented DNAs were used for library preparation with NEBNext^®^ DNA Sample Prep Reagent Set 1 (New England BioLabs, Ipswich, MA). The primers containing multiplexing index adapters (barcodes) to identify sequencing reads belonging to each sample were synthesized according to the specifications provided by Illumina Inc. for multiplex pair-end sequencing. Deep sequencing was performed at Macrogen (Seoul, Korea) using HiSeq2000 (Illumina) producing 101 bp-long paired-end reads, or at our laboratory using MiSeq (Illumina) producing 100 to 300 bp-long paired-end reads.

NEBNext mRNA Sample Prep Master Mix Set 1 (New England BioLabs, Ipswich, MA) was used for preparation of Illumina sequencing libraries from total RNA contained in the sample (whole-RNA library). Briefly, 0.5 μg total RNA (extracted as mentioned above) was used for fragmentation by focused ultrasonicator (Covaris) to generate the optimal fragment sizes of 200–300 bp suitable for RNA Illumina sequencing. The fragmented RNA was used for cDNA synthesis using ProtoScript II Reverse Transcriptase and random primers. The cDNA was further converted into double stranded cDNA with end-repair procedure (Klenow fragment, T4 polynucleotide kinase and T4 polymerase), and ligated to Illumina paired end (PE) adaptors. Size selection was performed using 2% agarose gel electrophoresis, generating cDNA libraries ranging from 275 to 325 bp in size. Finally, the libraries were expanded using 15 cycles of PCR with multiplex indexed primers and purified with magnetic beads using Agencourt Ampure Beads (Beckman Coulter).

### Data analysis

Reference RNA sequences of X-179A, X-181, 121XP, A/California/07/2009 (H1N1), and A/PR/08/34 viruses were downloaded from GenBank. Accession numbers for segments 1 to 8 of X-179A were CY058516 to CY058523 respectively. The same sequence references were used for X-181 except for segments 2, 4, and 6 were replaced by GQ906800—GQ906802 respectively. For 121XP virus, the reference sequences for segments 1 to 8 were GQ454863, GQ454864, GQ454865, GQ280121, GQ454866, GQ280122, GQ454867, and GQ454868 respectively. As references for A/PR/8/34 viruses, we used the sequences downloaded from GenBank; for segments 1–7 the accession numbers were EF467818- EF467824 respectively and EF467817 for segment 8. For A/California/07/2009 (H1N1) the following NCBI sequences were used for segments 1 to 8: CY121687, CY121686, CY121685, CY121680, CY121683, CY121682, CY121681, and CY121684 respectively.

The RNA sequences were used as references for alignment of the viral sequence reads generated in this study. First, sequencing reads with low quality (phred) scores were removed from the data set. The remaining sequences aligned with reference influenza virus sequences using custom software that is implemented in a highly integrated virtual environment (HIVE) computer cluster (https://hive.biochemistry.gwu.edu/dna.cgi?cmd=main).

To eliminate the false mutations, which could be an artifact produced during PCR amplification or sequencing procedures, the bias mutations were identified by Shannon entropy as a measure of randomness of distribution of mutations along individual sequencing reads [27]. Generally, a bias of mutation is considered significant if its entropy value is lower than one sigma (≈0.67). Also, only sequencing reads that have more than 75% of their expected size were aligned to the reference sequences, to eliminate nonspecific reads.

Finally, aligned sequencing reads were used to compute single-nucleotide polymorphism (SNP) profiles, read depth and the entropy for the entire viral genomes.

## Results

### Robustness of simultaneous influenza A genome segment amplification

To amplify all segments in one reaction, the reverse transcriptase Superscript III was used for cDNA synthesis, and two different PCR amplification protocols were used for DNA amplification.

**Fig 1:**
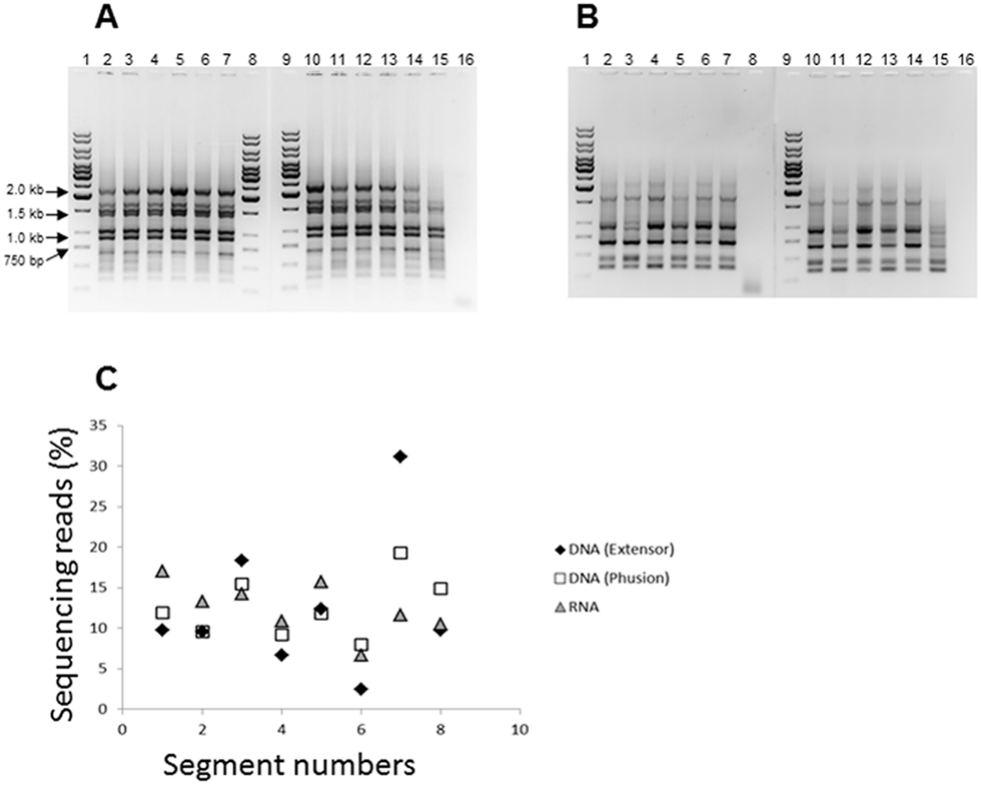
A: Simultaneous RT-PCR amplification of the eight segments of A/California/07/2009 (H1N1) vaccine viruses using Phusion DNA polymerase, 1: 1 kb DNA Ladder, 2: X-181-M1, 3: X-179A-M1, 4: X-181-M2, 5: X-179A-M2, 6: X-181-M3, 7: X-179A-M4, 8: 1 kb DNA Ladder, 9: 1 kb DNA Ladder, 10: 121XP-M4, 11: X-181-M4, 12: X-179A-M3, 13: X-179A-M5, 14: X-179A, 15: X-181, 16: Negative control (H_2_O). B: Simultaneous RT-PCR amplification of the eight segments of A/California/07/2009 (H1N1) vaccine viruses using Thermo Scientist Extensor Mix, 1: 1 kb DNA Ladder, 2: X-181-M1, 3: X-179A-M1, 4: X-181-M2, 5: X-179A-M2, 6: X-181-M3, 7: X-179A-M4, 8: Negative control (H_2_O), 9: 1 kb DNA Ladder, 10: 121XP-M4, 11: X-181-M4, 12: X-179A-M3, 13: X-179A-M5, 14: X-179A, 15: X-181, 16: Negative control (H_2_O). C: The distribution amount of sequencing reads between the eight segments of influenza virus using different approaches of library preparation followed by Illumina HiSeq sequencing.

The agarose gel electrophoresis results showed that all eight segments were amplified efficiently from passaged A/California/07/2009 (H1N1) pandemic vaccine viruses (The used viruses are presented in Table 1) by using Phusion DNA polymerase (New England BioLabs, Ipswich, MA) (Fig 1A). In contrast, use of Thermo Scientific Extensor Mix resulted in nonspecific bands and poor amplification of some segments (Fig 1B). To determine which of the two PCR protocols ensured unbiased amplification of all segments of influenza virus genome, the amplified samples (Fig 1A and 1B) were subjected to Illumina HiSeq sequencing. The percentage of sequencing reads obtained for each of the segments is presented in Fig 1C, along with the whole-RNA library that was run by HiSeq that was prepared as described in section 2.3 of the materials and methods paragraph. This result demonstrated that adequate distribution of sequencing reads between segments were obtained from a DNA library prepared from samples amplified by Phusion DNA polymerase (DNA library), and by whole-RNA library.

### Consistency of Illumina sequencing, and sequencing analysis of A/PR/8/34 and A/California/07/2009 strains

Center for Biologics Evaluation and Research (CBER) stock of A/PR/8/34 was kindly provided by Dr. Peter Palese at Mount. Sinai School of Medicine and was passaged in CBER to produce CBER stock, this virus was sequenced five times by MiSeq and HiSeq using whole-RNA library and DNA library approaches. The results of sequencing the RNA library showed high levels of reproducibility. There was a slight difference between MiSeq and HiSeq results only for mutations present at low levels (around 5%, Table 2). In contrast, the five repeats of DNA library sequencing were less consistent and more heterogeneous. (S1 Table).

**Table 2:** Percentage of mutations (≥5%) emerged in the genome of A/PR/8/34 strain (CBER stock) obtained by sequencing five times its RNA library by HiSeq and MiSeq. Notes: MiSeq values that differ from HiSeq's values are presented in parentheses. These mutation percentages were calculated per comparison to their published sequences. (See accession numbers in Materials and Methods paragraph.) nt: Nucleotide, aa: Amino-acid.

Segment	nt. Change	aa. Change	A-PR8-34 Repeat 1	A-PR8-34 Repeat 2	A-PR8-34 Repeat 3	A-PR8-34 Repeat 4	A-PR8-34 Repeat 5
PB2	279C→A	Silent (84Ala)	100	100	100	100	100
	306A→T	Silent (93Pro)	100	100	100	100	100
	1422C→A	Silent (465Pro)	100	100	100	100	100
	2127G→A	Silent (700Glu)	99	99	99	98	98
	2128A→G	701Asn→Asp	99	99	99	98	98
PB1	294G→A	Silent (90Glu)	100	100	100	100	100
	297G→A	Silent (91Ala)	100	100	100	100	100
	370C→A	116Gln→Lys				6 (<5)	5(<5)
	639G→A	205Met→Ile	100	100	100	100	100
	1002C→A	Silent (326Thr)	100	100	100	100	100
	1204T→C	394Ser→Pro	99	99	99	99	99
	1551T→C	Silent (509Leu)	99	99	99	99	99
	2076G→A	Silent (684Glu)	100	100	100	100	100
PA	323C→A	100Ala→Asp	5 (<5)	6 (<5)	5(<5)	6 (<5)	6(<5)
	323C→G	100Ala→Gly	5 (<5)	5(<5)	5(<5)	6 (<5)	6(<5)
	799C→A	259Pro→Thr	8 (5)	8 (6)	7 (5)		
	873A→G	Silent (283Leu)	100	100	100	100	100
	1233T→A	Silent (403Leu)	100	100	100	100	100
	2151A→C	Silent(709Ser)	99	99	99	99	99
HA	60T→A	10Cys→Ser	100	100	100	100	100
	1042A→C	337Asn→Thr	99(100)	99(100)	99	99(100)	99(100)
NP	54T→C	Silent(3Ser)	99(100)	99(100)	99	99(100)	99(100)
	69A→G	Silent(8Arg)	100	100	100	100	100
	282T→G	Silent (79Leu)	100	100	100	100	100
	630A→G	Silent (195Arg)	100	100	100	100	100
	924G→A	Silent (293Arg)	100	100	100	100	100
	1094C→A	350Thr→Lys	22 (18)	22 (18)	21 (18)	20 (18)	22 (18)
	1350G→A	Silent(435Gly)	100	100	100	100	100
NA	43T→C	8Ile→Thr	97	97	97	96	96
	258C→A	80Arg→Ser				5(<5)	5(<5)
	314C→A	98Asp→Glu	6 (<5)	6 (<5)	6 (<5)	7(<5)	7(<5)
	412G→A	131Ser→Asn	100	100	100	100	100
	773G→A	Silent(251Ser)	100	100	100	100	100
	860T→C	Silent(280Asn)	98 (99)	98(99)	98(99)	98(99)	98(99)
	1142G→T	Silent(374Val)	100	100	100	100	100
	1211G→A	Silent (397Leu)	100	100	100	100	100
	1357C→A	446Ala→Asp			5(<5)	5(<5)	5(<5)
M	67C→A	Silent (14Ile)	9(<5)	9(<5)	9(<5)	9(<5)	9(<5)
	136C→T	Silent (37Thr)	100	100	100	99%	100
	685A→G	Silent (220Gly)	100	100	100	100	100
	697C→A	224Ser→Arg	6 <5)	6 (<5)	7(<5)	6 (<5)	6 (<5)
	792A→G	5'UTR	100	99	99	99	99
NS	105C→A	27Leu→Ile	6 (<5)	6 (<5)	6 (<5)	7(<5)	6 (<5)
	170T→C	Silent (48Ser)	100	100	100	100	100
	173T→C	Silent (49Thr)	100	100	99	100	100
	189A→G	55Lys→Glu	100	100	100	100	99(100)
	575A→G	Silent (183Gly)	100	100	100	100	100

Most of A/PR/8/34 segments contained several complete mutations in comparison to their published sequences, and most of these mutations were silent. This virus was less heterogeneous, as few low substitutions were observed along the genome.

Another A/PR/8/34 virus stock was provided by National Institute for Biological Standards and Control (NIBSC) and was propagated 13 times in eggs and then was grown (passage 14) separately in two different eggs, and the resulting stocks were designated as A/PR/8/34-1 and A/PR/8/34-3 (Table 1). Both of them were used for whole-RNA library preparation, followed by HiSeq sequencing. The results in Table 3 showed that these two viruses shared almost all mutations, and the percentages of mutations were slightly higher in the A/PR/8/34-1 than in the A/PR/8/34-3 virus. They were more heterogeneous than CBER A/PR/8/34 virus stock, and all three A/PR/8/34 viruses shared several complete mutations along their genomes (Tables 2 and 3). They also shared one low-level mutation C_1094_A (Thr_350_Lys) in the NP gene which was present in about 20% of the RNA sequences in all three A/PR/8/34 viruses.

**Table 3:** Percentage of mutations (≥5%) emerged in A/PR/8/34 viruses 1 and 3 genomes that were subjected to RNA library preparation followed by HiSeq sequencing. Note: These mutation percentages were calculated per comparison of A/PR/8/34 strains 1 and 3 genomes with the corresponding published sequences. (See accession numbers in Materials and Methods paragraph.) nt: Nucleotide, aa: Amino-acid.

Segment	nt. Change	aa. change	A/PR/8/34-1	A/PR/8/34-3
PB2	51A→G	Silent (8Arg)	28	12
	130A→C	35Thr→Pro	15	
	133T→G	36Ser→Ala	17	5
	139A→T	38Arg→Stp	6	
	142C→A	39Gln→Lys	6	5
	1083G→A	Silent (352Leu)	5	
	1306C→A	Silent(427Arg)		6
	2128A→G	701Asn→Asp	99	99
	2142G→C	Silent(705Gly)		6
	2149C→T	Silent (708Leu)	10	
	2199G→A	Silent (724Val)	15	6
	2238G→A	Silent (737Arg)	36	14
	2243G→A	739Arg→Gln	23	9
PB1	115A→T	31Ser→Cys	10	5
	133G→A	37Gly→Arg	20	10
	179A→G	52Lys→Arg	18	9
	189G→A	55Trp→Stp	19	11
	192A→T	Silent (56Thr)	13	7
	370C→A	116Gln→Lys		6
	2076G→A	Silent (684Glu)	33	16
	2097G→A	Silent (691Arg)	37	19
	2145A→G	Silent (707Arg)	40	21
	2178G→A	718Met→Ile	36	18
	2179G→T	719Val→Phe	35	16
	2236G→A	738Glu→Lys	46	23
	2267C→A	748Ser→Tyr	6	7
PA	150G→A	Silent (42Leu)	6	
	323C→A	100Ala→Asp	7	8
	323C→G	100Ala→Gly	7	8
	873A→G	Silent (283Leu)	99	99
	903G→A	Silent (293Glu)	5	5
	2151A→C	Silent (709Ser)	99	98
HA	724C→A	231Ala→Glu		5
	1042A→T	337Asn→Ile	100	100
	1052T→C	Silent (340Ile)	9	5
	1413C→A	461Leu→Met	5	5
NP	132C→T	Silent (29Val)	8	
	184C→A	47Leu→Ile		6
	1094C→A	350Thr→Lys	19	22
NA	258C→A	80Arg→Ser	5	6
	304C→A	95Ser→Tyr		6
	773G→A	Silent (251Ser)	100	100
	1357C→A	446Ala→Asp	7	6
M	58A→T	Silent (11Val)	23	9
	67C→A	Silent (14Ile)	6	9
	100A→G	Silent (25Ala)	24	11
	113G→A	30Asp→Asn	28	11
	130G→A	Silent (35Lys)	30	13
	240G→A	72Arg→Gln	26	10
	241A→G	Silent (72Arg)	29	11
	334C→T	Silent (103Leu)	27	11
	353C→T	110His→Tyr	25	10
	361C→T	Silent (112Ala)	29	11
	370C→A	Silent (115Ile)	26	11
	371T→G	116Ser→Ala	25	11
	385T→C	Silent (120Ser)	22	9
	401A→T	126Ser→Cys	25	9
	434G→A	137Ala→Thr	23	10
	442C→T	Silent(139Thr)	25	9
	443A→G	140Thr→Ala	28	10
	453C→T	143Ala→Val	22	8
	454A→G	Silent (143Ala)	23	10
	524A→G	167Thr→Ala	26	12
	643T→G	Silent (206Ala)	23	10
	645G→T	207Ser→Ile	22	9
	648A→G	208Gln→Arg	25	10
	655A→G	Silent (210Arg)	27	11
	667A→G	Silent (214Gln)	27	11
	670G→A	Silent (215Ala)	29	12
	685A→G	Silent (220Gly)	100	100
	697C→A	224Ser→Arg	7	8
	716A→G	231Asn→Asp	24	10
	792A→G	5'UTR	31	11
	793C→T	5'UTR	22	9
	801G→T	5'UTR	29	11
	805A→G	5'UTR	26	10
	829C→T	5'UTR	22	9
	874G→T	5'UTR	27	9
	887A→T	5'UTR	27	12
	888C→T	5'UTR	25	9
	894G→A	5'UTR	26	10
	943G→A	5'UTR	31	12
NS	591G→A	189Asp→Asn	6	7
	616C→A	197Thr→Asn		6

A/California/07/2009 (H1N1) strain was sequenced by HiSeq using the whole-RNA library approach, and the summary of the results is presented in Table 4. No complete substitutions were observed, and the highest percentage of mutants observed was 66% for mutation A_57_G (Glu_18_Gly) in the PA segment. No mutations were observed in the NA segment. Three low-level non-synonymous mutations were found in HA: A_456_G (Asn_146_Asp, 11%), A_736_G (Asp_239_Gly, 16%) and G_739_A (Arg_240_Gln, 16%). The Asn_146_Asp mutation is located in the Sa antigenic site.

**Table 4:** HiSeq sequencing of RNA Library prepared from A/Ca/07/2009 (H1N1) virus. Note: These mutation percentages were calculated per comparison of A/California/07/2009 (H1N1) genome with its published sequence. (See accession numbers in Materials and Methods paragraph.) aa: Amino acid, nt: Nucleotide.

Segment	nt. Change*	aa. Change	Mutation percent (≥5%)
PB2	114G→T	33Lys→Asn	7
	125G→C	37Gly→Ala	5
	129G→A	Silent (38Arg)	5
	1726C→T	Silent (571Leu)	46
PA	57A→G	18Glu→Gly	66
	121C→A	39Cys→Stp	7
HA	456A→G	146Asn→Asp	11
	736A→G	239Asp→Gly	16
	739G→A	240Arg→Gln	16
NP	335G→A	101Gly→Asp	31
	337G→A	102Gly→Arg	5
	357C→T	Silent (108Leu)	6
	374A→G	114Glu→Gly	6
	1156G→A	375Asp→Asn	15

*: Count of nucleotide location started 20 nucleotides (UTR) before the starting codon AUG.

### 3.3. Deep sequencing analysis of 121XP virus passaged in eggs

The 121XP virus was generated by the reverse genetics method followed by serial passages in chicken eggs; this virus contains the HA and NA genes from A/California/07/2009 (H1N1) and all other genes from A/PR/8/34 (H1N1) [26].

To evaluate its genetic stability, the 121XP virus was passaged ten times in eggs under manufacture conditions (Table 1). Mutations revealed by deep sequencing in HA and NA genes of the virus are summarized in Table 5. Several non-synonymous mutations emerged in NA and HA, including the mutations Asn_16_Ser and Lys_226_Glu that were present at low levels (36% and 18% of mutants, respectively). The latter mutation is located in the Ca antigenic site in HA. Mutation Lys_136_Asn, located close to the antigenic site Sa, emerged and accumulated to about 78% of the population.

**Table 5:** Mutation percentages (≥5%) emerged in HA and NA of 121XP-M4 virus passaged in eggs. Note: These mutation percentages were calculated per comparison with the corresponding expanded wild-type progenitor. (See accession numbers in Materials and Methods.) aa: Amino-acid, nt: Nucleotide.

Segment	nt. Change	aa. Change	HiSeq		MiSeq
			DNA-Library	RNA-Library	RNA-Library
HA	47A→G	16Asn→Ser	32	36	33
	338T→C	113Ile→Thr	7	7	7
	408G→T	136Lys→Asn	79	78	78
	561G→A	Silent (187Gly)	9	10	9
	676A→G	226Lys→Glu	15	18	16
NA	166T→C	56Tyr→His	14	17	17
	248T→C	83Val→Leu	14	13	16
	255A→G	85Leu→Val	84	83	82
	262A→G	88Asn→Asp	83	81	80
	263A→G	88Asn→Asp	84	83	81
	264T→C	88Asn→His	14	17	19
	355A→G	119Lys→Glu	100	100	100
	708T→G	Silent (236Gly)	37	37	38
	1352A→G	Silent (451Asp)	35	42	38
	1354A→G	452Thr→Ala	35	47	39

Ten mutations were found in the NA gene, and eight of them led to amino acid changes. Interestingly, three of them were found in codon 88. Sequencing of other genomic segments revealed several heterogeneities in most of them, with some mutations leading to amino acid changes. There was a good agreement between results obtained by sequencing of the whole-RNA library by MiSeq and HiSeq, as well as the DNA library by HiSeq. For more details, see S2 to S5 Tables.

### Deep sequencing analysis of X-179A viruses passaged in eggs

**Fig 2:**
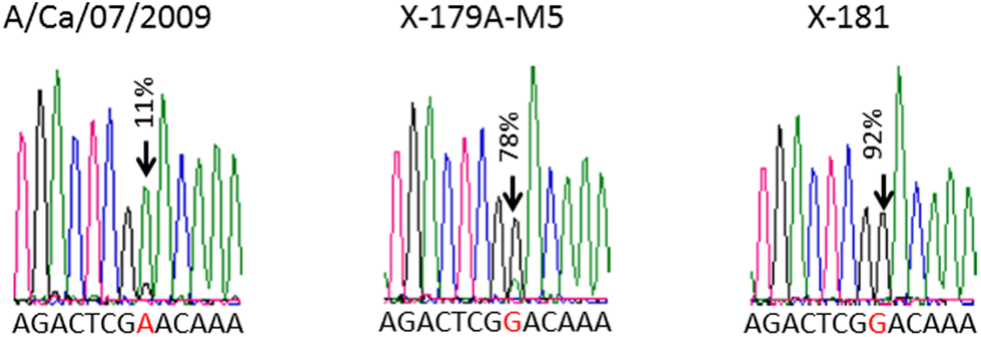
Sanger sequencing of the presence of mutation Asn_146_Asp (A_436_G) that was detected by Illumina sequencing at diffent levels: 11% in A/Ca/07/2009 (H1N1) virus, 78% in X-179A-M5 virus and 92% in X-181 virus.

The X-179A virus is a reassortant prepared by New York Medical College (NYMC) using classical reassortant methodology from A/California/7/2009 (H1N1) virus and NYMC X-157 virus, with the HA, NA and PB1 genes originating from A/California/7/2009 (H1N1), and the other internal genes from A/PR/8/34 (H1N1) [26]. This virus was passaged several times and adapted to grow in eggs. Viruses derived by passaging of X-179A virus in chicken eggs under manufacture conditions (Table 1) were similarly analyzed by deep sequencing. The results of deep sequencing of HA and NA genes of these viruses are summarized in Table 6. In sample X-179A-M5, two non-synonymous mutations emerged in HA: Pro_314_Gln (17%) and Asn_146_Asp (78%). The latter mutation located in the Sa antigenic site was present as low level mutation in A/California/07/2009 (H1N1). The confirmation of the presence of this mutation by Sanger sequencing method is presented on Fig 2. Sample X-179A-M2 contained a low level (9%) of Lys_328_Thr mutation.

**Table 6:** Mutation percentages (≥5%) emerged in HA and NA of X-179A viruses passaged in eggs. Notes: The percentages obtained from RNA library followed by HiSeq sequencing are presented without parentheses or brackets. The percentages obtained from RNA library followed by MiSeq sequencing are presented in parentheses. The percentages obtained from DNA library followed by HiSeq sequencing are presented in brackets. These mutation percentages were calculated per comparison with the corresponding expanded progenitor. (See accession numbers in Materials and Methods.) aa: Amino-acid, nt: Nucleotide.

Segment	nt. Change	aa change	X-179A	X179A-M1	X-179A-M2	X-179A-M3	X-179A-M4	X-179A-M5
HA	436A→G	146Asn→Asp						78 (82) [80]
	941C→A	314Pro→Gln						17 (16)[16]
	983A→C	328Lys→Thr			9(8)[8]			
NA	284G→T	95Ser→Ile				7(7)[7]		
	911T→G	304Val→Gly	[5]					

One non-synonymous mutation, Ser_95_Ile, was present at a low level (7%) in NA of sample X179A-M3.

In general, samples derived from X-179A virus were heterogeneous and contained several complete substitutions compared to its published sequence.

HiSeq sequencing of DNA libraries prepared from X-179A viruses revealed more heterogeneity than HiSeq/MiSeq sequencing of RNA libraries prepared from the same samples. Mutations found in other genomic segments are presented in S5 to S7 Tables.

### Deep sequencing analysis of X-181 viruses passaged in eggs

Similar to X-179A virus, X‐181 virus was developed by New York Medical College using conventional reassortment technology. It has a 5:3 gene constellation with three genes (PB1, HA and NA) coming from A/California/07/2009 (H1N1) and the remaining five genes from A/PR/8/34 [26]. The A/PR/8/34 genes were provided by reassorting NYMC X‐157 with A/California/07/2009 (H1N1). This virus is produced to grow well in eggs, and was passaged several times in eggs.

Viruses derived by passaging of the X-181 virus in chicken eggs under manufacture conditions (Table 1) were analyzed by deep sequencing. The results of deep sequencing of HA and NA genes of these viruses are summarized in Table 7. Two mutations were found in HA genes: A_1398_G (silent, 31%) in the X-181-M3 virus and G_756_T (Glu_252_Asp, 47%) in the X-181-M4 virus. The latter mutation is located in the conserved region of the antigenic site Ca. One non-synonymous mutation, Thr_466_Ala, was present at a low level (8%) in the NA gene of X-181-M2. Interestingly, the mutation Asn_146_Asp is located in the Sa antigenic site and exists as a complete substitution in X-181 virus which is derived from X-179A virus. As mentioned above, this mutation was observed in X-179A-M5 at a high level (78%) and at a low level (11%) in the wild-type A/California/07/2009 (H1N1) virus.

**Table 7:** Mutation percentages (≥5%) emerged in HA and NA of X-181 viruses passaged in eggs. Notes: The percentages obtained from RNA library followed by HiSeq sequencing are presented without parentheses or brackets. The percentages obtained from RNA library followed by MiSeq sequencing are presented in parentheses. The percentages obtained from DNA library followed by HiSeq sequencing are presented in brackets. These mutation percentages were calculated per comparison with the corresponding expanded progenitor. (See accession numbers in Materials and Methods.) aa: Amino-acid, nt: Nucleotide.

Segment	nt. change	aa. Change	X-181	X-181-M1	X-181-M2	X-181-M3	X-181-M4
HA	436G→A	146Asp→Asn	(8)				
	756G→T	252Glu→Asp					47(46)[46]
	1398A→G	Silent(466Val)				31(31)[29]	
NA	465T→C	Silent(155Tyr)				5	
	911T→G	304Val→Gly	[7]				
	1396A→G	466Thr→Ala			20(6)[8]		

Viruses derived by passaging of X-181 in chicken eggs were heterogeneous and contained several complete mutations in PB2, NP and NS segments (S8 to S10 Tables).

As for X-179A viruses, HiSeq sequencing of DNA libraries prepared from X-181 viruses revealed more heterogeneity than HiSeq/MiSeq sequencing of whole-RNA libraries prepared from the same samples. Mutations found in other genomic segments are presented in S8 to S10 Tables.

## Discussion

Rapid emergence of mutations is an inherent property of RNA viruses, leading to genetic heterogeneity of viral populations [8]. This allows viruses to rapidly adapt to changing growth conditions by selecting from the preexisting variants with higher fitness [28]. For some vaccines, virus growth in cell cultures during vaccine manufacture leads to reversion of attenuated phenotype to virulence; therefore, their content must be monitored as a part of vaccine quality control [13]. Mutations emerging during virus growth may also alter the antigenic properties, and as a consequence affect protective potency of live and inactivated vaccines. Antigenic properties of influenza A are notoriously prone to rapid changes. Hemagglutinin (HA) and neuraminidase (NA) are the two most important influenza virus envelope proteins that elicit immune response, and therefore are under immunological pressure that leads to their continuous change. The antigenic drift reflects the accumulation of amino acid substitutions in the globular domain of HA, the principal target of neutralizing antibodies, as well as in NA [29, 30]. The primary function of HA is the attachment to host cell receptors and mediation of viral entry [31, 32]. The NA is involved in progeny virion release and spread of the virus, and also may have a role in HA-mediated membrane fusion [33, 34]. Adaptation of viruses to replication in new cell types (e.g. growth in cell substrates, such as embryonated eggs or mammalian cells used for vaccine manufacture) involves changes in HA. Since the receptor-binding region on the HA molecule overlaps with the major antigenic site, the adaptation may also be accompanied by changes in antigenic properties [33, 35, 36].

The larger number of eggs used during manufacture process would decrease the level (percentage) of each individual mutant, but could nevertheless increase the overall heterogeneity of virus population. Cumulatively, they could lead to a similar loss of antigenic properties as one mutation present at a higher level. Therefore, it may be important to monitor the changes that take place in major protective epitopes of the seed virus stocks to avoid losses in protective efficacy of the vaccine. Not all mutations that emerge in viruses are harmful to vaccine properties. Mutations that consistently accumulate have higher fitness and could increase virus yields and vaccine potency. Therefore, studying mutations emerging in vaccine strains could be used for both quality control, and for optimization of the genetic composition of vaccine strains.

Seed viruses used in influenza vaccine manufacture are produced either by reassortment technology that uses co-cultivation of a high-growth reference strain with the current virus isolate leading to exchange of genomic segments [37], or by reverse genetics based on transfection of susceptible cells with a combination of cDNA plasmids coding for the appropriate segments of the virus [38]. Both approaches have their strengths and weaknesses, and can potentially lead to the emergence of mutants. One of the purposes of this study was to determine which of them produces more homogeneous virus stocks with a lower number of mutants. We have used three pandemic influenza A/California/07/2009 (H1N1) virus vaccine strains and virus stocks produced from these viruses by passaging them on eggs. X-179A was prepared at the New York Medical College on eggs as a reassortant between A/California/07/2009 and A/PR/8/34. After reports of a low yield of production virus from X-179A a second reassortant, X-181, was made. X-181 contains the HA, NA and PB1 segments from A/California/07/2009. 121XP was made using reverse genetics followed by serial passages in eggs and only has the HA and NA segments from A/California/07/2009 virus [26].

To reveal sequence heterogeneity in viral stocks we have used a deep sequencing approach that was recently shown to be an effective tool for quantifying all mutants in viral populations [24]. Mutations that were identified by deep sequencing and were present at a significant level were also confirmed by the analysis of Sanger sequencing electrophoregrams that revealed sequence heterogeneities. Amplification of the entire genome of influenza viruses presents a challenge because it consists of eight genomic segments of different lengths and sequences. Previously, we described PCR conditions that allowed us to amplify all genomic segments of influenza A virus in one reaction [27]. In this work, the PCR amplification was optimized to amplify all influenza segments simultaneously, efficiently and equally, which resulted in an adequate distribution of sequencing reads between all segments (Fig 1A and 1C). We have also used whole-RNA libraries prepared without specific amplification of viral cDNA. Illumina deep-sequencing of viral cDNA libraries and whole-RNA libraries were used to determine quantitative profiles of mutations along the entire genome of viruses of influenza A/California/07/2009 (H1N1) vaccines.

The deep sequencing results revealed several heterogeneities in most genomic segments, and several mutations led to amino acid changes. No big differences were observed between the results obtained by sequencing of whole-RNA libraries using MiSeq and HiSeq, and HiSeq sequencing of DNA libraries (S2 to S10 Tables). Deep sequencing of whole-RNA libraries was found to be more reproducible than sequencing of DNA libraries (Table 2). But the HiSeq sequencing of DNA libraries were less consistent and produced more heterogeneous sequences (S1 Table). This may be due to errors introduced by PCR amplification and non-specific alignment of primers.

Deep sequencing of several stocks of high-growth reference strain A/PR/8/34 revealed also some mutations and sequence heterogeneities. The CBER master stock of this strain contained several complete mutations in comparison to the published sequence, and most of these mutations were silent. There were also low levels of additional mutations. This indicates that this A/PR/8/34 virus is genetically stable, and the use of this virus as backbone for development of influenza vaccine may improve the vaccine production.

Another A/PR/8/34 virus stock was passaged 13 times on eggs and was grown (passage 14) separately in two different embryonated chicken eggs, the harvested 2 viruses (designated as A/PR/8/34-1 and A/PR/8/34-3) were deep-sequenced using HiSeq protocol. These two viruses shared most sequence heterogeneities, but the percentages of mutants were slightly different (Table 3). Therefore, virus growth in eggs introduced some additional sequence heterogeneity, but caused no complete nucleotide substitutions.

The parental wild type A/California/07/2009 (H1N1) virus was sequenced by HiSeq using the whole RNA library (Table 4). Its consensus matched the published nucleotide sequence. There was some sequence heterogeneity observed at select positions, reaching up to 66% (Glu_18_Gly) of the viral population, this mutation is very rare and nothing known about its biological function.

The Deep sequencing of the X-179A passaged viruses revealed several mutations in HA and NA. Two non-synonymous mutations in HA (Pro_314_Gln present at 17% and Asn_146_Asp present at 78% of the population) were identified in X-179A-M5 virus. Interestingly, the latter is located in the antigenic site Sa; it was detected at a low level (11%) in A/California/07/2009 (H1N1) strain and was present as a complete substitution in the X-181 virus stock derived from the X-179A virus (Fig 2), suggesting that it may be involved in the adaptation of X-181 virus. Virus X-179A-M2 contained Lys_328_Thr mutation at a low level (9%). Viruses derived from X-179A were heterogeneous and contained some complete nucleotide substitutions in comparison to their published sequences in PB2, PB1, NP, and in NS segments (see S5 to S7 Tables).

The X-181 virus was developed from the X-179A seed lot by another round of reassortment, and also is subjected to several passages in eggs (Table 1). Deep sequencing results showed that the G_756_T (Glu_252_Asp, present at 47%) mutation emerged in HA of the X181-M4 virus, and it is located in the conserved region of the antigenic site Ca.

Unlike the two previous viruses, 121XP was developed by reverse genetics [26]. Deep sequencing of virus derived from it showed several non-synonymous mutations in NA and HA. Mutations leading to Asn_16_Ser and Lys_226_Glu amino acid substitutions emerged at relatively low levels (36% and 18% of mutants, respectively) in HA. The latter mutation was located in the Ca antigenic site in HA, next to codon 225 that was shown to participate in the modulation of HA receptor avidity/specificity [39]. In addition, mutation of residue 225 was found to enable H3 viruses to switch from avian to human specificity [40, 41]. Mutation Lys_136_Asn was present at a high level (78%). It is located close to the antigenic site Sa in close proximity within the sialic acid-binding pocket, suggesting its functional importance [42]. Interestingly, in the NA segment, three mutations occurred in codon 88, suggesting that this codon represents a hot spot. Also, the results of passages 10 showed that the overall number of mutations and sequence heterogeneities present in virus stocks derived by reverse genetics was higher than the number of mutations in virus stocks created by conventional reassortment procedure. This observation is counterintuitive, since reverse genetics is based on homogeneous cDNA plasmids coding individual viral segments. One possible explanation for increased sequence heterogeneity of cDNA-derived RNA viruses is that transcription of cDNA plasmids to rescue the virus followed by its passages to adapt to grow in chicken eggs led to higher sequence heterogeneity.

In conclusion, our deep sequencing approach based on RNA library preparation was shown to be effective and reproducible for detection of low quantities of mutants in the entire genome of influenza viruses. It revealed that the viruses derived from the three of pandemic A/Ca/07/2009 (H1N1) vaccine viruses have varying levels of sequence heterogeneities. It can be used for assessment of the quality of seed viruses, and for ensuring that they do not contain mutations that could affect immunogenic properties of the vaccine.

## Supporting Information

S1 Table Percentage of mutations (≥5%) emerged in the genome of A/PR/8/34 strain (CBER stock) obtained by sequencing five times its DNA library by HiSeq. (XLS)

S2 Table Percentage of mutations (≥5%) emerged in 121XP-M4 virus obtained by using RNA library preparation followed by MiSeq sequencing. (XLS)

S3 Table Percentage of mutations (≥5%) emerged in 121XP-M4 virus obtained by using RNA library preparation followed by HiSeq sequencing. (XLS)

S4 Table Percentage of mutations (≥5%) emerged in 121XP-M4 virus obtained by using DNA library preparation followed by HiSeq sequencing. (XLS)

S5 Table Percentage of mutations (≥5%) emerged in X-179A viruses obtained by using RNA library preparation followed by MiSeq sequencing. (XLS)

S6 Table Percentage of mutations (≥5%) emerged in X-179A viruses generated by using RNA library preparation followed by HiSeq sequencing. (XLS)

S7 Table Percentage of mutations (≥5%) emerged in X-179A viruses generated by using DNA library preparation followed by HiSeq sequencing. (XLS)

S8 Table Percentage of mutations (≥5%) emerged in X-181 viruses generated by using RNA library preparation followed by MiSeq sequencing. (XLS)

S9 Table Percentage of mutations (≥5%) emerged in X-181 viruses generated by using RNA library preparation followed by HiSeq sequencing. (XLS)

S10 Table Percentage of mutations (≥5%) emerged in X-181 viruses generated by using DNA library preparation followed by HiSeq sequencing. (XLS)
