# Correction: Deep Sequencing for Evaluation of Genetic Stability of Influenza A/California/07/2009 (H1N1) Vaccine Viruses

**DOI:** 10.1371/journal.pone.0142248

**Published:** 2015-11-02

**Authors:** The PLOS ONE Staff

The first author’s first and last names are incorrectly switched. The publisher apologizes for this error. The correct name is: Majid Laassri. The correct author byline is: Majid Laassri, Tatiana Zagorodnyaya, Ewan P. Plant, Svetlana Petrovskaya, Bella Bidzhieva, Zhiping Ye, Vahan Simonyan, Konstantin Chumakov. The correct citation is: Laassri M, Zagorodnyaya T, Plant EP, Petrovskaya S, Bidzhieva B, Ye Z, et al. (2015) Deep Sequencing for Evaluation of Genetic Stability of Influenza A/California/07/2009 (H1N1) Vaccine Viruses. PLoS ONE 10(9): e0138650. doi:10.1371/journal.pone.0138650
